# In vitro antibacterial and cytotoxic activity of leaf extracts of *Centella asiatica* (L.) Urb*, Warburgia salutaris* (Bertol. F.) Chiov and *Curtisia dentata* (Burm. F.) C.A.Sm - medicinal plants used in South Africa

**DOI:** 10.1186/s12906-018-2378-3

**Published:** 2018-11-29

**Authors:** Oluwagbemiga Sewanu Soyingbe, Nkoana Ishmael Mongalo, Tshepiso Jan Makhafola

**Affiliations:** 1 Research, Innovation and Engagements Portfolio, Mangosuthu University of Technology, Durban, 4031 South Africa; 2 University of South Africa, College of Agriculture and Environmental Sciences Laboratories, Private Bag X06, Florida, Johannesburg 0710 South Africa

**Keywords:** Medicinal plants, Ethno-medicine, Antibacterial activity, Immunosuppressed, Anti-proliferative effect

## Abstract

**Background:**

Compounds having both anticancer and antimicrobial activity have promising therapeutic potential due to their selective cytotoxicity and their potential to reduce the occurrence of bacterial and fungal infections in immune-compromised cancer patients. In our quest to find new antimicrobial agents with potent anticancer activity, the biological potential of leaves from the three medicinal plants *Centella asiatica*, *Warburgia salutaris* and *Curtisia dentata* as used by Zulu traditional healers for the treatment of cancer is investigated.

**Methods:**

Extracts were assayed for antibacterial activity using the agar well diffusion and micro plate dilution assay. In addition, minimum bactericidal concentrations (MBC), lactate dehydrogenase (LDH) release assay and rhodamine 6G intake assay were used to ascertain the antibacterial activity. The cytotoxic effects of the plant extracts were determined using tetrazolium-based colorimetric (MTT) cell proliferation assay against MCF-7, human colorectal carcinoma cells (Caco-2), A549 and HeLa cancerous cell lines.

**Results:**

The acetone extracts from *Waburgia salutaris* revealed noteworthy anti-proliferative effect yielding IC_50_ value of 34.15 μg/ml against MCF-7 cell line, while acetone extract from *Curtisia dentata* significantly (*P* ≤ 0.05) revealed promising IC_50_ values of 41.55, 45.13, 57.35 and 43.24 μg/ml against A549, HeLa, CaCo-2 and MCF-7 cell lines. The extracts further revealed a broad-spectrum antibacterial activity against bacterial strains used in the study. An acetone extract from *W. salutaris* revealed the highest zone of inhibition and the lowest minimum inhibitory concentration (MIC) of 21.0 mm and 0.16 mg/ml respectively against *Staphylococcus aureus.* Methanol extract from *W. salutaris* and ethyl acetate extract from *C. dentata* revealed 53% inhibition of R6G inside the cell against *Staphylococcus aureus* and *Escherichia coli* respectively in a cytosolic lactate dehydrogenase assay, suggesting that the mode of action of such extracts may be through efflux pump.

**Conclusions:**

Overall, the extracts had good antibacterial activity and anti-proliferative effects against selected cancerous cell lines. Given the good antibacterial activity of the extracts the plants may act as an immune booster and prevent infection in immunosuppressed cancer patients. This is further supported by the plants’ anti-proliferative potential, bacteriostatic, bactericidal properties and also their ability to block bacterial efflux pump systems.

## Background

Chemopreventive agents from medicinal plants are being sought after as a promising approach to control and manage life-threatening human infections like cancer [1, 2]. Studies of various medicinal plants and other natural products from nature have shown the anti-proliferative, cytostatic and cytotoxic activities of various phytochemicals against different cancer cells [3]. Traditional African medicine is amongst the most ancient natural therapies and the oldest form of medicine currently used. Combined with western drugs, it can be used to produce one of the most successful regimes to treat a variety of illnesses [4]. Traditional African medicine is based on using botanical preparations to treat human and animal illness. Such medicine is prepared by extracting roots, barks, leaves, flowers, seeds from, or the entire plant of, different plant species [5]. Different types of plants contain a variety of phytochemicals, which may well act as antioxidants, antimicrobial, anti-inflammatory and anti-cancer agents, making the use of plants more advantageous [6]. Furthermore, these plants generally possess little or no side effects when compared to western medicine. Immunosuppression is the suppression of the immune system’s ability to fight infection. This is often caused by diseases like HIV/AIDS, lymphoma and cancer. Immunosuppression is a major complication in cancer and also an adverse effect of chemotherapy during the treatment of cancer. Most patients being treated with broad-based chemotherapy develop immunosuppression. Chemotherapy treatment is intended to target rapidly dividing cells. Unfortunately, it does not discriminate between cancer cells and other rapidly dividing cells. The body’s immune system cells are equally rapidly dividing and may unfortunately become the target of chemotherapy. This invariably exposes patients receiving chemotherapy to infection. The most common clinical manifestation of immunosuppression is infection, which may be bacterial, viral, or fungal [7–10]. *Centella asiatica* (L.) Urb, *Warburgia salutaris* (Bertol. f.) Chiov and *Curtisia dentata* (Burm. F.) C.A.Sm are African medicinal plants which are generally used by traditional healers for the treatment and management of cancer [11–14]. *C. asiatica* is from the Araliaceae family and its leaves are used for the treatment of fever, rheumatoid arthritis and skin related disorders [13–15]. *W. salutaris* is from the Canellaceae family and used in the treatment of malaria, venereal diseases, cancer, coughs and colds [11, 16, 17]. *C. dentata* belongs to the Cornaceae family and its stem, bark and leaves are used in the treatment of cancer, stomach disorders, diarrhoea, sexually transmitted diseases and as a blood purifier [14, 18].

In the current study, we evaluate the antibacterial activities of these medicinal plants, as used in the treatment of cancer by traditional healers in South Africa against nosocomial pathogenic bacterial strains due to the fact that the most common clinical manifestation of immunosuppression is bacterial infection.

## Method

### Plant materials

The leaves of *C. asiatica*, *W. salutaris* and *C. dentata* were collected from the South African National Biodiversity Institute (SANBI) Lowveld Botanical Garden in Nelspruit, South Africa. The voucher specimens of *C. asiatica*, *W. salutaris* and *C. dentata* were prepared and matched with voucher specimen numbers Glow 12/2011, Glow 167/1988 and Glow 96/1998 respectively within Lowveld Herbarium. The plant materials were identified by Willem Froneman the resident horticulturist.

### Extraction

The plant leaves were dried at room temperature and powdered using a hammer mill (Ika Scientific, Germany) to yield approximately 2 mm mesh size powder. The leaves of each plant were soaked (1:10 *w*/*v*) in methanol, ethyl acetate, dichloromethane and distilled water respectively in 200 ml Schott Duran bottles. The bottles were placed in in mechanical shaker (IKA Scientific, Model MF 10 B, Germany) at 100 rpm for 48 h to extract the phytochemicals. The extracts were filtered through a Whatman No.1 filter paper, and then concentrated and dried using a Buchi rotoary evaporator. The water extracts were dried using a bench top freeze dryer (Labconco Corporation, Kansas City, Moussouri, USA) after being frozen at 20 °C. All the solvents used were of AR grade and obtained from Merck, South Africa.

### Microorganisms

A total of fourteen microorganisms were selected and used in the study. The organisms were collected from the University of South Africa, College of Agriculture and Environmental Sciences Laboratories. *Enterococcus avium (*ATCC 14025), *Staphylococcus aureus* (ATCC 6538), *Streptococcus agalactiae* (ATCC 12386), *Bacillus cereus* (ATCC 10702), *Enterococcus hirae* (ATCC 10541), *Enterococcus faecalis* (Clinical isolate), *Enterococcus gallinarium* (ATCC 49573), *Escherichia coli* (ATCC 25922), *Pseudomonas aeruginosa* (ATCC 10031), *Proteus mirabilis* ATCC (29906), *Proteus vulgaris* ATCC (49132), *Klebsiella pneumoniae* (ATCC 13883), *Acinetobacter calcaoceuticus anitratus* (CSIR) and *Salmonella typhi* (ATCC 14028) were selected for the study. The stock cultures were maintained at 4 °C in Müeller-Hinton agar (Oxoid, Germany).

### Antibacterial assay

#### Agar disk diffusion

The study of antibacterial activity of the selected medicinal plant extracts was carried using the agar disk diffusion method [19]. Bacterial strains were grown overnight at 37 °C separately in 10 ml nutrient broth. The bacteria cultures were then diluted to the McFarland no.5 standard (1.0 × 10^8^ CFU/ml). Bacteria suspension (1.0 × 10^8^ CFU/ml) were then inoculated with into standard Petri dishes with nutrients agar. Sterile paper disks of 6 mm (Mast Diagnostics, Germany) were placed on the inoculated plates after which the paper disk were soaked with 10 μl of 10 mg/ml of the extracts in 1% DMSO. Tests were performed in triplicates. Zones of inhibition were measured in mm as a diameter in areas where no growth of the bacteria was visible.

#### The minimum inhibitory concentration (MIC)

The MIC of the extracts was determined using the micro plate dilution assay [20]. Nutrients broth (50 μl) was added into all the wells of the 96 well microtitre plate. About 50 μl of all the extracts (5 mg/ml) in 1% DMSO was then serially diluted down the rows from row of the 96 well microtitre plate. Bacterial culture (50 μl) of McFarland standard was then added to all the wells of the 96 well microtitre plate then incubated at 37 °C for 24 h. *p*-Iodo-nitrotetrazolium violet (INT) solution (40 μl of 0.2 mg/ml) was then added into all the well of the 96 well microtitre plate and incubated at 37 °C for 30 min. The MIC was defined as the lowest concentration with no visible microbial growth observed within the plate.

#### The minimum bactericidal concentration (MBC)

Minimum bactericidal concentration is defined as the lowest concentration of the plant extract where all the inoculated bacterial strains are completely killed. Culture medium 10 μl from the microtiter plates used for MIC was reinoculating, on nutrient agar plates and incubated at 37 °C for 24 h. [21].

#### Lactate dehydrogenase (LDH) release assay (membrane damage)

The cytosolic LDH release assay was done using microplate technique previously described [22–25]. The LDH release assay was calculated with the formula: (E-C)/(T-C) × 100, where E is the absorbance of the experimental cell cultures, C is the absorbance of the cell medium used as the control, and T the absorbance of the positive control which is the LDH release of Triton X-100 lysed cells.

#### Rhodamine 6G uptake

The extracts were tested for their Efflux pump inhibitory activity using the method of Maesaki et al. (1999) [26] with some modifications as described by Soyingbe et al. (2015) [50]. The percentage accumulation of R6G inside cells after exposure to glucose, essential oil and standards was calculated from the formula:

$$ \mathrm{Percentage}\ \mathrm{accumulation}\ \mathrm{of}\ \mathrm{R}6\mathrm{G}=\left(1-{\mathrm{A}}_{\mathrm{t}}/{\mathrm{A}}_{\mathrm{o}}\right)\ \mathrm{x}\ (100) $$

Where A_t_ is the absorbance of the test compound and A_o_ is the absorbance of the control in the presence of glucose only.

#### Cytotoxity assay

Human breast adenocarcinoma (MCF-7) cells, human cervical cancer cells (HeLa), human embryonic kidney cells (HEK293) and human colorectal carcinoma cells (Caco-2) were grown to confluency in 75 cm^3^ flasks. The cells were then trypsinised and plated into 96 well plates at specific seeding densities of 23 × 10^4^ cells per well, incubated overnight at 37 °C and 5% CO_2_. The medium was then removed and fresh medium (MEM + Glutmax + antibiotics+ 10% Fetal bovine serum) was added. Extracts were then added in triplicate and incubated for 48 h. At the end of the incubation period, test samples were removed from the wells and replaced with 200 μl fresh medium to which 30 μl of 5 mg/ML MTT was added and incubated for 4 h. After incubation with MTT, the medium in each well was removed and the formazan crystals that formed were dissolved by adding 50 μL of DMSO to each well of the plates. The plates were gently shaken until the crystals dissolved. The amount of MTT reduction was measured immediately by detecting the absorbance at 570 nm [27]. The percentage of cell viability was calculated using the formula below:

$$ \%\mathrm{Cell}\ \mathrm{viability}=\mathrm{Mean}\ \mathrm{absorbance}\ \mathrm{of}\ \mathrm{sample}/\mathrm{Mean}\ \mathrm{absorbance}\ \mathrm{of}\ \mathrm{control}\ \mathrm{X}\ 100 $$

The IC_50_ values (lethal concentration at which 50% of the cells are killed) were calculated from linear regression plots as the concentration of the test sample that resulted in 50% reduction of absorbance compared to untreated cells. The intensity of the MTT formazan produced by living metabolically active cells is directly proportional to the number of live cells present.

### Statistical analysis

The results of each assay was analyzed using Graphpad Prism 6, data were expressed as means ± SD of triplicate measurements. *P* values ≤0.05 were referred to as statistically significant.

## Results

The antibacterial activity of the plant extracts against 14 bacterial strains, is shown in Table 1. In the agar diffusion assay, the selected medicinal plants showed a broad-spectrum antibacterial activity against both gram positive and gram negative organism with zones of inhibition ranging from 7 mm to 29 mm. The methanol extracts of *C. asiatica* and *C. dendata*, had the highest antibacterial activity yielding 10 mm to 19 mm. The inhibitory zone of 8 mm to 21 mm was observed for the acetone extract of *W. salutaris*. The minimum inhibitory concentration (MIC) activity of methanol, ethyl acetate acetone and water leaf extracts of *C. asiatica*, *W. salutaris* and *C. dentata* are presented in Table 2. The MIC values of the extracts ranged from 0.31 mg/ml to >10 mg/ml. The methanol extract of *W. salutaris* revealed a lowest MIC value of 0.31 mg/ml against *E. avium* and *E. coli*, while the acetone extracts had an MIC value of 0.31 mg/ml on *E.coli.* The MIC value of 0.31 mg/ml for the methanol extract of *C. dentata* on *Staphylococcus aureus* was also noted. The cytosolic lactate dehydrogenase release which measures the membrane damage is shown in Table 3. Only extracts that showed MBC activities were tested for their LDH release activity. The highest cytosolic lactate dehydrogenase release ranged from 0 to 36.4% release. The accumulation of R6G in the cells after exposure to glucose, plant extracts and standard inhibitor berberine are presented in Table 3. Insofar as the LDH activity was concerned, only extracts with MBC values were tested further for their efflux pump inhibitory activity. The value of the drug accumulation in the presence of glucose alone was taken as the control 0%. The accumulation range was from 0 to 53%, with the ethyl acetate extract of *C. dentata* showing a high efflux pump inhibitory capacity. The results of the cytotoxicity investigations are presented in Table 4 and the cytotoxic effects of the plant extracts were tested against 4 cell line. The IC_50_ values of the extracts, which is the lethal concentration at which 50% of the cells are killed (calculated as the average from three independent experiments) against doxorubicin hydrochloride as a reference drug, are represented in Table 4. *C. asiatica* acetone extract showed the most significant activity, with IC_50_ value of 53.65 ± 0.06 μg/ml against MCF-7 and 46. 49 ± 0.04 μg/ml for A549. The water extracts were the least active and showed IC_50_ value of >100 μg/ml for MCF-7, Caco-2 and A549 and IC_50_ value of 76.3 ± 0.06 μg/ml for HeLa. The acetone extracts from *C. dentata* and *W. salutaris* revealed the most active cytotoxic effect. Acetone extracts from *C. dentata* exhibited IC_50_ values of 43.24 ± 0.01 and 41.55 ± 0.04 μg/ml against MCF-7 and A549 respectively, while the acetone extract from *W. salutaris* revealed IC_50_ values of 34.15 ± 0.77 and 44.51 ± 1.25 μg/ml against A549 and MCF-7 respectively. The methanol and ethyl acetate extracts from *C. dentata* revealed IC_50_ values ranging from 53.27 ± 0.76 μg/ml to 82.6 ± 0.13 μg/ml against the four selected cell lines.

**Table 1:** Antibacterial activities of Leaf extracts of *Centella asiatica, Warburgia salutaris* and *Curtisia dentate*

Zone 0f inhibition														
	Centella Asiatica				Curtisia Dentate				Warburgia salutaris				Controls	
Test organisms	Methanol	Ethyl Acetate	Acetone	water	Methanol	Ethyl Acetate	Acetone	water	Methanol	Ethyl Acetate	Acetone	water	Cipro	1% DMSO
*E. avium*	19 ± 2.0	12 ± 0.4	13 ± 2.0	10 ± 0.4	18 ± 1.5	11 ± 2.0	15 ± 0.6	7 ± 0.1	16 ± 0.6	11 ± 2.1	14 ± 3.0	11 ± 1.1	31 ± 2.1	NA
*S. aureus*	18 ± 1.4	13 ± 1.4	10 ± 0.7	7 ± 3.0	15 ± 2.5	13 ± 1.5	17 ± 1.0	11 ± 1.1	19 ± 2.0	12 ± 2.0	21 ± 1.0	8 ± 1.2	27 ± 0.5	NA
*S. agalactiae*	29 ± 0.4	10 ± 2.5	15 ± 0.3	8 ± 1.2	12 ± 1.5	15 ± 1.2	16 ± 1.5	11 ± 0.2	13 ± 1.2	9 ± 1.2	14 ± 0.5	NA	22 ± 1.5	NA
*B. cereus*	19 ± 0.0	10 ± 0.4	9 ± 1.2	8 ± 0.6	19 ± 1.5	13 ± 0.6	13 ± 1.0	10 ± 0.8	13 ± 1.2	10 ± 0.6	15 ± 1.0	9 ± .0.9	25 ± 0.5	NA
*E. hirae*	15 ± 1.0	13 ± 0.4	11 ± 1.4	8 ± 2.8	13 ± 0.7	15 ± 2.5	14 ± 1.2	9 ± 1.5	17 ± 1.5	12 ± 0.8	11 ± 0.5	7 ± 1.5	28 ± 1.5	NA
*E. faecalis*	16 ± 0.4	21 ± 0.4	13 ± 2.2	10 ± 3.1	18 ± 1.5	13 ± 1.2	14 ± 1.5	11 ± 0.2	12 ± 2.1	10 ± 0.5	12 ± 0.2	8 ± 0.6	35 ± 1.7	NA
*E. gallinarium*	10 ± 0.5	14 ± 1.7	11 ± 1.0	7 ± 0.3	16 ± 2.0	12 ± 1.2	12 ± 1.0	7 ± 1.4	13 ± 0.6	12 ± 0.3	10 ± 1.0	NA	26 ± 2.0	NA
*E. coli*	16 ± 0.0	8 ± 1.0	10 ± 0.3	8 ± 1.1	10 ± 1.4	11 ± 1.4	11 ± 1.5	NA	12 ± 1.0	10 ± 1.2	14 ± 1.5	10 ± 1.4	21 ± 1.7	NA
*P. aeruginosa*	17 ± 1.0	8 ± 0.6	12 ± 1.0	NA	12 ± 0.7	12 ± 0.6	12 ± 1.0	NA	14 ± 1.0	9 ± 1.5	12 ± 0.6	8 ± 1.0	23 ± 1.5	NA
*P. mirabilis*	14 ± 1.0	10 ± 1.6	9 ± 1.1	8 ± 0.0	10 ± 1.4	15 ± 0.4	15 ± 0.5	9 ± 2.5	16 ± 0.6	NA	11 ± 0.7	NA	26 ± 1.1	NA
*P. vulgaris*	9 ± 1.0	9 ± 0.6	12 ± 0.6	9 ± 0.8	16 ± 2.5	14 ± 0.3	11 ± 0.8	7 ± 3.0	11 ± 0.0	9 ± 0.6	8 ± 0.4	NA	24 ± 0.5	NA
*K. pneumonia*	12 ± 2.0	7 ± 0.6	10 ± 0.6	NA	10 ± 2.1	11 ± 1.5	10 ± 1.5	NA	12 ± 1.0	11 ± 2.0	11 ± 1.0	NA	25 ± 2.0	NA
*Acinetobacter* *Calcaoceuticals* *Anitratus*	10 ± 0.6	8 ± 0.3	9 ± 0.7	NA	12 ± 2.3	11 ± 1.1	10 ± 0.6	8.0 ± 1.4	13 ± 1.0	10 ± 1.2	10 ± 1.1	NA	23 ± 1.5	NA
*S. typhi*	11 ± 1.1	9 ± 0.6	7 ± 0.6	NA	12 ± 0.7	10 ± 1.5	12 ± 0.6	NA	13 ± 1.5	11 ± 1.5	9 ± 1.2	NA	27 ± 0.5	NA

Inhibition zone diameters (mm) including diameter of the sterile disc (6 mm): values are given as mean ± SD (3 replicates). ND = not determined; NA = not active. DMSO = Dimethyl sulfoxide. SD = standard deviation. (*P* ≤ 0.05)

**Table 2:** The MIC (mg/ml) values of *Centella asiatica*, Curtisia dentate and Warbugia salutaris extracts against bacteria

	Centella Asiatica				Curtisia Dentate				Warburgia salutaris				Controls	
Test organisms	Methanol	Ethyl Acetate	Acetone	water	Methanol	Ethyl Acetate	Acetone	water	Methanol	Ethyl Acetate	Acetone	water	Cipro	1% DMSO
*E. avium*	2.5	5	2.5	>10	0.63	5	0.63	5	0.31	1.25	1.25	10	0.63	ND
*S. aureus*	2.5	1.25	1.25	5	0.31	2.5	0.63	5	1.25	1.25	0.16	5	0.16	ND
*S. agalactiae*	5	5	5	>10	0.63	5	1.25	>10	5	>10	1.25	5	2.5	ND
*B. cereus*	2.5	2.5	1.25	5	0.63	5	1.25	5	1.25	5	1.25	10	0.63	ND
*E. hirae*	2.5	5	1.25	>10	1.25	5	5	5	1.25	2.5	2.5	5	5.0	ND
*E. faecalis*	5	10	5	10	>10	>10	5	>10	>10	>10	5	>10	0.63	ND
*E. gallinarium*	1.25	5	2.5	5	1.25	5	5	>10	2.5	2.5	5	5	0.31	ND
*E. Coli*	1.25	5	0.63	>10	0.63	2.5	1.25	10	0.31	2.5	0.31	5	0.31	ND
*P. aeruginosa*	5	10	5	10	1.25	>10	0.63	>10	2.5	>10	2.5	10	1.25	ND
*P. mirabilis*	2.5	>10	5	>10	0.63	5	1.25	>10	2.5	5	2.5	>10	0.31	ND
*P. vulgaris*	5	5	2.5	10	5	5	2.5	10	>10	5	5	10	1.25	ND
*K. pneumonia*	>10	>10	5	>10	>10	>10	5	>10	2.5	5	5	>10	5.0	ND
*Acinetobacter* *Calcaoceuticals* *Anitratus*	5	>10	>10	>10	5	>10	>10	>10	5	>10	2.5	>10	5.0	ND
*S. typhi*	>10	>10	>10	>10	5	>10	5	>10	5	>10	>10	>10	2.5	ND

MIC values given as mg/ml, results are mean of three replicates, *ND* = not determined, *DMSO* = Dimethyl sulfoxide; *Cipro* = Ciprofloxacin. (*P* ≤ 0.05)

## Discussion

Natural products which are obtained from medicinal plants, extracted in their crude form or as purified isolates, have been used as bases for discovering new drugs because of their chemical diversity. Interest and demand are on the rise for the chemical diversity applied in screening programs. The quest for therapeutic drugs obtained from natural products, particularly edible plants, has grown throughout the world [28, 29]. In this study we investigated the antibacterial and cytotoxicity properties of 3 important medicinal plants used in the treatment of cancer by traditional healers in South Africa against nosocomial pathogenic bacteria. Treatments using herbal medicines provide some advantages over the use of single purified chemicals [15, 30]. This could be because herbal medicines are mixtures of different therapeutic or preventive compounds or components, and could provide more activity in treating diseases than single products on their own [4, 31]. The dried plant leaves materials were extracted using different solvents, ethyl acetate, acetone, methanol and water. Water being the preferred solvent used by most traditional healers because of its availability. However, water does not extract non-polar bio-active compounds. Hence, in our study we decided to use other solvents to extract all compounds of varying polarities. The methanol and acetone extracts were the most active, as compared to the ethyl acetate and water extracts. These findings are similar to those of other authors [32] who reported that the methanol leaf extract of *C. asiatica* has moderate antibacterial activity against *Staphylococcus aureus*, *Bacillus cereus*, *Pseudomonas aeruginosa* and *Escherichia coli*, while the acetone extract was less effective against these same organisms. In other studies [33] the methanol extract of *C. asiatica* leaves revealed the highest inhibitory activity when compared to the acetone and aqueous extracts. Additionally, *Waburgia ugandensis* which had a similar composition pattern of its essential oil with *W. salutaris*, inhibited gram positive bacteria *S. aureus* and *Streptococcus pnemoniae* which were the most sensitive to the extract [34].

Elsewhere, *W. salutaris* showed high antibacterial activity against test bacteria and its methanol extract was the only extract that showed activity against *Escherichia coli*, a gram negative organism [35]. Higher antimicrobial activity of methanol extracts from *W. salutaris* could be related to sesquiterpene dialdehydes which are known to have antibacterial and cytotoxic activities and previously isolated from other taxonomically related *Waburgia* species [36]. Muzigadial was also identified as the main antibacterial agent in the stem bark of *W. salutaris* [16], with MIC of 12.5 μg/ml against both *S. aureus* and *B. subtilis* similar to the present study. Results of antimicrobial potentials of *C. dentata* against *E. coli* serotypes ranged between 8 and 28% which is similar to this present study similarly other authors [37, 38] reported MIC values using *C. dentata* methanol extracts that agree with the present study [39]. The positive control ciprofloxacin was also active against all the 14 organisms tested, whilst 1% DMSO and the negative control i.e. sterile distilled water were both not active on any of the 14 organisms tested.

The MBC values shown in Table 5 ranged between 2.5 mg/ml and 10 mg/ml. The lowest MBC value of 2.5 mg/ml was observed in the methanol and acetone extracts of *W. salutaris* on *E. coli*, while only the acetone extract of *W. salutaris* had an MBC value of 2.5 mg/ml on *S. aureus*. The water extracts showed no bactericidal activities and are therefore not shown.

The highest release of 36.4% was revealed by the acetone extract of *W. salutaris* on *S. aureus* (Table 3). This could be attributed to the fact that gram positive organisms possess a single layer cell membrane, and more susceptible to antibiotics compared to gram negative organisms, that have a double membrane and are less susceptible to the effect of antimicrobial agents [40]. Although most studies show that essential oils disrupt the permeability barrier of bacteria cell membranes [41]. The results of the membrane damage activity in this study show a low membrane damage activity similar to that reported by other authors [42]. The LDH release activity values of the extracts are generally low which shows that the mode of action of the extracts are not through membrane damage of the bacteria cells.

**Table 3:** Antibacterial percentage cytosolic lactate dehydrogenase assay and percentage accumulation of R6G inside the cell after exposure to glucose, plant extract and standard inhibitor bererine

Percentage accumulation and release														
		*Centella asiatica*				Curtisia dentate				Warburgia salutaris				Control
Test organisms		Methanol	Ethyl Acetate	Acetone	Water	Methanol	Ethyl Acetate	Acetone	water	Methanol	Ethyl Acetate	Acetone	water	berberine
*E. avium*	LDH^a^	0.68	–	0	–	8.1	–	0.7	–	2.4	–	0	–	53
	R6G^b^	11	31	10	–	30	–	24	–	3	–	21	–	
*S. aureus*	LDH^a^	0	0.3	3.6	–	7.2	2.1	5.4	–	0.8	0	36.4	–	87
	R6G^b^	20	42	5	–	9	35	25	–	53	20	6	–	
*S. agalactiae*	LDH^a^	–	–	–	–	–	–	0.7	–	0.5	–	1.4	–	76
	R6G^b^	–	–	–	–	–	–	14	–	15	–	3	–	
*B. cereus*	LDH^a^	–	0.8	4.3	–	1.4	–	–	–	15.1	–	0.3	–	85
	R6G^b^	–	39	13	–	12	–	–	–	10	–	8	–	
*E. hirae*	LDH^a^	–	–	–	–	–	–	–	–	–	0	0.1	–	75
	R6G^b^	–	–	–	–	–	–	–	–	–	24	9	–	
*E. faecalis*	LDH^a^	–	–	–	–	–	–	–	–	–	–	1.0	–	67
	R6G^b^	–	–	–	–	–	–	–	–	–	–	6	–	
*E. gallinarium*	LDH^a^	0	–	–	–	–	–	5.1	–	1.6	–	–	–	55
	R6G^b^	9	–	–	–	–	–	18	–	23	–	–	–	
*E. Coli*	LDH^a^	0.8	–	18.6	–	1.4	0	18.6	–	25.4	1.34	4.3	–	92
	R6G^b^	11	–	45	–	31	53	39	–	35	24	11	–	
*P. aeruginosa*	LDH^a^	–	–	–	–	–	–	4.4	–	3.3	–	0.9	–	64
	R6G^b^	–	–	–	–	–	–	51	–	24	–	7	–	
*P. mirabilis*	LDH^a^	–	–	–	–	0.7	–	–	–	–	–	–	–	41
	R6G^b^	–	–	–	–	18	–	–	–	–	–	–	–	
*P. vulgaris*	LDH^a^	–	–	0	–	–	–	–	–	–	–	–	–	50
	R6G^b^	–	–	6	–	–	–	–	–	–	–	–	–	
*K. pneumonia*	LDH^a^	–	–	–	–	–	–	–	–	4.7	0	–	–	79
	R6G^b^	–	–	–	–	–	–	–	–		6	–	–	
*Acinetobacter* *Calcaoceuticals* *Anitratus*	LDH^a^	–	–	–	–	–	–	–	–	–	–	0	–	66
	R6G^b^	–	–	–	–	–	–	–	–	–	–	23	–	
*S. typhi*	LDH^a^	–	–	–	–	–	–	–	–	–	–	0	–	45
	R6G^b^	–	–	–	–	–	–	–	–	–	–	11	–	

^a^LDH release (membrane damage) activity % LDH Release in comparison to Triton X-100; ^b^Values of drug accumulation in the presence of glucose were taken as the control, − = not determined. (*P* ≤ 0.05)

**Table 4:** Cytotoxicity activity of methanol, Acetone and Ethyl Acetate of plant extracts

	*Centella asiatica*				*Curtisia dentata*				*Warburgia salutaris*				Control
Cells	Methanol	Ethyl Acetate	Acetone	water	Methanol	Ethyl Acetate	Acetone	water	Methanol	Ethyl Acetate	Acetone	water	Dox
MCF-7	77.6 ± 0.12	49.36 ± 0.04	53.65 ± 0.06	> 100	82.6 ± 0.13	61.38 ± 0.05	43.24 ± 0.01	76.7 ± 0.04	59.57 ± 1.23	44.59 ± 0.70	34.15 ± 0.77	> 100	1.26 ± 0.03
CACO-2	> 100	> 100	81.88 ± 1.35	> 100	78.56 ± 0.31	74.71 ± 1.25	57.35 ± 0.04	> 100	> 100	92.23 ± 0.15	73.29 ± 0.16	> 100	2.76 ± 0.06
HELA	78.30 ± 1.60	74.75 ± 0.24	56. 88 ± 0.72	76.3 ± 0.06	61. 30 ± 0.33	59.49 ± 0.04	45.13 ± 0.03	98.7 ± 1.25	84. 00 ± 1.25	71.27 ± 0.09	56. 01 ± 0.38	77.5 ± 0.11	1.43 ± 0.24
A549	68.51 ± 0.25	63.73 ± 0.16	46.49 ± 0.04	> 100	67.35 ± 0.33	53.27 ± 0.76	41.55 ± 0.04	> 100	52.51 ± 0.24	56.72 ± 1.92	44.51 ± 1.25	> 100	2.10 ± 0.11

IC_50_ values of the Cytotoxicity of plant extracts on different cancer cell lines MCF-7, CACO-2, HeLa and A549. DOX = Doxorubicin hydrochloride. Data expressed as mean ± standard deviation, *n* = 3. (P ≤ 0.05)

**Table 5:** The MBC (mg/ml) values of *Centella asiatica*, Curtisia dentate and Warbugia salutaris extracts against bacteria

	Centella Asiatica				Curtisia Dentate				Warburgia salutaris				Controls	
Test organisms	Methanol	Ethyl Acetate	Acetone	water	Methanol	Ethyl Acetate	Acetone	water	Methanol	Ethyl Acetate	Acetone	water	Cipro	1% DMSO
*E. avium*	5	>10	10	>10	2.5	>10	10	>10	5	>10	10	>10	0.63	ND
*S. aureus*	10	5	5	>10	5	10	5	>10	10	10	2.5	>10	0.16	ND
*S. agalactiae*	>10	>10	>10	>10	>10	>10	10	>10	10	>10	5	>10	2.5	ND
*B. cereus*	>10	10	2.5	>10	10	>10	>10	>10	5	>10	5	>10	0.63	ND
*E. hirae*	>10	>10	>10	>10	>10	>10	>10	>10	>10	10	10	>10	5.0	ND
*E. faecalis*	>10	>10	>10	>10	>10	>10	>10	>10	>10	>10	10	>10	0.63	ND
*E. gallinarium*	10	>10	>10	>10	>10	>10	10	>10	10	>10	>10	>10	0.31	ND
*E. Coli*	5	>10	5	>10	10	5	5	>10	2.5	5	2.5	>10	0.31	ND
*P. aeruginosa*	>10	>10	>10	>10	>10	>10	10	>10	10	>10	10	>10	1.25	ND
*P. mirabilis*	>10	>10	>10	>10	5	>10	>10	>10	>10	>10	>10	>10	0.31	ND
*P. vulgaris*	>10	>10	10	>10	>10	>10	>10	>10	>10	>10	>10	>10	1.25	ND
*K. pneumonia*	>10	>10	>10	>10	>10	>10	>10	>10	10	10	>10	>10	5.0	ND
*Acinetobacter* *Calcaoceuticals* *Anitratus*	>10	>10	>10	>10	>10	>10	>10	>10	>10	>10	10	>10	5.0	ND
*S. typhi*	>10	>10	>10	>10	>10	>10	>10	>10	>10	>10	>10	>10	2.5	ND

MBC values given as mg/ml, results are mean of three replicates ND = not determined, DMSO = Dimethyl sulfoxide; Cipro = Ciprofloxacin. (*P* ≤ 0.05)

The efflux pump confers bacterial resistance [43]. The development of new antibiotics which act as efflux pump inhibitors (EPIs) is very important in order to reduce the emergence of multidrug resistance (MDR) to the present antibiotics being used. Recent studies have shown that medicinal plants have bioactive compounds (such as tannins, alkaloids, flavonoids etc) responsible for antibacterial properties [44]. The accumulation of R6G was species specific, as observed in the current study. These results were in accordance with results found by other authors [45], which showed that R6G concentration are increase with plant extracts. *S. aureus* was noted to have the highest percentage accumulation when compared with other organisms.

In a study carried out by Babu et al., 1995 [46], purified fractions of *C. asiatica* showed anti-proliferative effects having IC_50_ values of 17 μg/mL and 22 μg/mL against Ehrlich ascites tumor cells, which agrees with our study. On the other hand, Pittella et al. 2009 [47] showed aqueous extracts of *C. asiatica* to reveal no anti-proliferative activity against mouse melanoma (B16F1), human breast cancer (MDA MB-231) and rat glioma (C6) cell lines with IC_50_ value of 698.0 μg/mL, 648.0 μg/mL and 1000.0 μg/mL respectively. Betulinic acid was found to be the most abundant of the isolated compounds, present in most of the bulk fractions of *C. dentata* [38]. Betulinic acid was selectively cytotoxic against several human melanoma cancer cell lines. It was also found to be active in vivo against athymic mice carrying human melanoma with little toxicity [48]. The mechanism of action of betulinic acid on mammalian cells is thought to involve the induction of apoptosis [48]. By the standard set by National Cancer Institute, a crude extract is said to have anti-tumor properties when exhibiting the IC_50_ value of less than 50 μg/ml [49], suggesting that the extracts from the selected medicinal plants revealed some anti-cancer effects against the selected cell lines.

## Conclusion

It is imperative that more drugs be discovered to combat the upsurge of cancer. Medicinal plants are being sought after as the potential way forward in treating cancer and also the underlying effects of cancer treatments. The methanol extracts of *C. Dentate* and *W. Salutaris* showed the highest antibacterial activity while the acetone extract of *W. Salutaris* showed the most promising cytotoxicity effect. Given the good antibacterial activity of the extracts the plants may act as an immune booster and prevent infection in immunosuppressed cancer patients. This may also be due to their bacteriostatic and bactericidal activities, and also their ability to block bacterial efflux pump systems. With the plant extracts showing a good overall antibacterial activity and moderate cytotoxic effect in this study, the results of this study suggest that the different extracts have the potential to be exploited as a good source for the development of antimicrobial agents.

## Abbreviations

LDH: Lactate Dehydrogenase
MBC: Minimum bactericidal concentration
MIC: Minimum inhibitory concentration
R6G: Rhodamine 6G
